# Pulmonary embolism after dexamethasone treatment for COVID-19: a case report

**DOI:** 10.1186/s12879-022-07228-2

**Published:** 2022-03-22

**Authors:** Hidenori Takahashi, Yoshinobu Iwasaki, Takayasu Watanabe, Naoki Ichinose, Toshimi Oda

**Affiliations:** 1 Department of Pulmonary Medicine, Showa General Hospital, 8-1-1 Hanakoganei, Kodaira, Tokyo 187-8510 Japan; 2 Department of Infection Control, Showa General Hospital, Kodaira, Japan

**Keywords:** COVID-19, SARS-CoV-2, Pulmonary embolism, Anticoagulation, Dexamethasone, Cytokine

## Abstract

**Background:**

Although the RECOVERY trial showed that dexamethasone was efficacious for the treatment of coronavirus disease 2019 (COVID-19), its impact on the risk of pulmonary embolism (PE) and other serious procoagulant events was not assessed.

**Case presentation:**

Here we report the case of a previously healthy 83-year-old woman with COVID-19, without any genetic predisposition to thrombosis. She developed moderate respiratory distress 12 days after symptom onset and a 10-day course of dexamethasone therapy was initiated. Her clinical condition and imaging findings improved initially; however, they deteriorated after the completion of dexamethasone therapy, despite the improvement in her pneumonia and viral clearance. Laboratory tests showed markedly raised serum D-dimer, ferritin, and sIL-2R levels, and contrast-enhanced computed tomography showed deep vein thrombosis (DVT) in the left iliac vein and PE of the right pulmonary artery. The DVT and PE were successfully treated using intravenous heparin administration.

**Conclusions:**

This case illustrates the potential risk of rebound inflammation and procoagulant events following dexamethasone withdrawal. We believe that COVID-19-induced DVT and PE can be affected by dexamethasone therapy. Although dexamethasone reduces procoagulant factors, increases anticoagulant factors, and modulates cytokines, which can suppress/delay thrombus formation during treatment, it confers the risk for rebound cytokine production after treatment completion, triggering cytokine and coagulation cascades that can lead to thromboembolic diseases. In this critical clinical period, the patient’s deteriorating condition may be overlooked because of the masking effects of dexamethasone treatment on fever and other clinical conditions and laboratory changes. Clinicians should follow-up coagulation markers carefully and contrast-enhanced computed tomography is useful for detecting coagulation; and, if PE occurs, therapeutic heparin administration is essential because emboli can also generate cytokines.

## Background

Coronavirus disease 2019 (COVID-19), caused by severe acute respiratory syndrome coronavirus 2 (SARS-CoV-2), was first reported in December 2019 in Wuhan, China, and has rapidly spread worldwide. As of November 22, 2020, the World Health Organization reported over 57.8 million cases and 1.3 million deaths, with mortality rates continuing to rise [1]. Furthermore, the COVID-19 crisis in the Northern hemisphere during the winter of 2020–2021 will be unpredictable as it may worsen due to SARS-CoV-2’s preference for cold and dry conditions and indoor environments [2, 3].

Although COVID-19 primarily manifests as a respiratory tract infection, SARS-CoV-2 may also cause systemic infection accompanied by coagulation disorders. This may progress to fatal procoagulant events, including deep vein thrombosis (DVT) and pulmonary embolism (PE) in patients with COVID-19 [4]. The clinical management of COVID-19 coagulopathies is difficult due to its unpredictable occurrence. It typically develops 7–17 days after symptom onset, particularly in patients with severe disease, but some patients experience late occurrence, even after COVID-19 has improved. In patients with non-severe disease, it is often overlooked [4, 5]. In patients with COVID-19, a hyperinflammatory state driven by a cytokine storm contributes to the development of DVT and PE, possibly akin to that of secondary hemophagocytic lymphohistiocytosis (HLH) [6, 7].

The RECOVERY trial found that dexamethasone is an effective therapy for COVID-19 and reduces the duration of ventilation and mortality [8]. However, its impact on procoagulant events has not been assessed. It is generally thought that low-dose corticosteroids reduce procoagulant factors, increase anticoagulant factors [9], and modulate plasma levels of proinflammatory cytokines, which may play important roles in COVID-19, similar to that in HLH coagulopathies [10, 11].

There have been no previous reports of DVT and PE related to dexamethasone use in patients with COVID-19. The presence of PE may be overlooked due to difficulties in diagnosis. PE clinical manifestations include fever, dyspnea, and hypoxemia, which can be indistinguishable from COVID-19 symptoms [12]. Although it may be more challenging to diagnose and treat PE occurring in patients with COVID-19 during/after dexamethasone therapy, it is urgent that clinical management strategies be developed.

In this case report, we describe a patient with COVID-19 who developed DVT and PE and deteriorated after receiving dexamethasone therapy. DVT and PE resolved after continuous intravenous heparin infusion.

## Case presentation

**Fig. 1:**
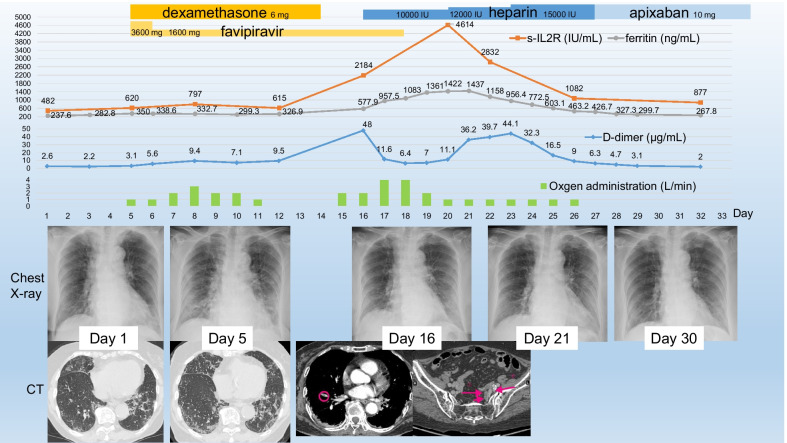
Clinical course of the patient. Chronological changes in ferritin and soluble interleukin-2 receptor (top) and D-dimer (mid upper) levels, along with changes in oxygen dosage (mid-lower) and imaging findings (bottom)

A previously healthy, active 83-year-old female presented with a 7-day history of persistent mild fever, and was diagnosed with COVID-19 after a positive real-time loop-mediated isothermal amplification test result and admitted to our hospital in early October. She was a non-smoker and had no history of autoimmune disease or congenital coagulation disorders. On admission, physical examination revealed a body temperature of 37.2℃ and oxygen saturation of 96% on room air. Laboratory tests showed normal hepatic and renal function but coagulation abnormalities, and computed tomography (CT) found bilateral ground glass opacities with a predominantly peripheral location (Fig. 1). She only required isolation and did not require oxygen administration during the first four days in hospital.

On day 5 post-admission, her respiratory condition worsened. She developed moderate respiratory distress and required oxygen administration. Both chest radiography and CT revealed the presence of shadows. We administered oral dexamethasone (6 mg/day on days 5–14) and favipiravir (two 1800 mg doses on day 5, followed by 800 mg twice daily on days 6–18) [8].

On day 8 she required an oxygen intake of 3  L/min via nasal cannula, which was followed by a gradual improvement of her respiratory condition and chest imaging. Oxygen administration was discontinued on day 12. During this period, laboratory findings, including serum D-dimer, ferritin, and soluble interleukin-2 receptor (sIL-2R) levels, were stable. Prophylactic anticoagulant therapy was not initiated because her condition was improving and she was mobile [13].

On day 16, two days after completing the dexamethasone course, she developed fever, oxygen desaturation, and a reduced level of consciousness, despite continual improvement of her chest X-ray findings. Laboratory tests showed markedly raised serum D-dimer, ferritin, and sIL-2R levels. Contrast-enhanced CT scan showed PE of the right pulmonary artery and DVT of the left iliac vein up to the level of the bifurcation of the common iliac vein. Continuous intravenous heparin infusion at a dose of 10,000–15,000 IU/day was initiated, with a target of maintaining an activated partial thromboplastin time of 1.5–2.5 times the normal level [13].

On day 18, her oxygen requirement peaked at 4 L/min, administered via nasal cannula, followed by improvement in her condition. Her serum sIL-2 and ferritin levels peaked on days 20 and 21, followed by a decline in her D-dimer levels. Nasopharyngeal transcription-reverse transcription concerted reaction (TRCR) tests (TRCReady, Tosoh Bioscience, Japan) were negative on days 19, 23, and 27, and chest X-rays on days 21 and 30 revealed bilateral regression of the shadows in the lower lung fields. Oxygen administration was discontinued on day 27. Heparin was replaced with oral apixaban (5 mg twice daily), and she was discharged on day 33.

## Discussion and conclusions

In this patient, we encountered COVID-19-induced DVT and PE, which may have been affected by dexamethasone therapy. Her coagulation status and serum cytokine levels were assessed using markers such as serum D-dimer, ferritin, and sIL-2R, which also serve as prognostic markers in COVID-19 and are important diagnostic markers of HLH [10, 14]. HLH is a fatal hyperinflammatory syndrome that is triggered by infections, malignancies, immune checkpoint inhibitors, or autoimmune disorders. It is associated with cytokine storms, which require a balanced treatment approach that stops the underlying triggers and neutralizes the inflammatory cytokines [10, 15].

Notably, these markers appeared to be suppressed during dexamethasone therapy and increased after dexamethasone was withdrawn, remaining high for approximately 10 days despite a clinical improvement in COVID-19 and undetectable SARS-CoV-2 using TRCR. This raises the clinical question as to why the patient experienced a second deterioration and had hypercytokinemia for such a long period, despite improvement of her pneumonia and viral clearance. SIL-2R, which reflects T lymphocytes activated by IL-2, has a half-life of only 6 h in vivo [16]. We believe that continuous cytokine production was the core pathophysiology underlying her second deterioration.

SARS-CoV-2 in endothelial cells may cause endothelial damage, which can trigger the release of proinflammatory cytokines, including IL-2, that activate the coagulation cascade and consequently, a cytokine storm resulting in blood coagulation and intravascular microthrombosis, which promote the development of DVT and PE [4]. Additionally, intravascular microthrombi induced by COVID-19 may form neutrophil extracellular traps (NET)-containing neutrophil-platelet infiltrates, which can be a secondary source of cytokines through activation of neighboring macrophages [6].

In our patient, dexamethasone therapy may have suppressed cytokine production [11], while favipiravir administration reduced the SARS-CoV-2 viral load [17]. These therapies may have contributed to the improvement in the patient’s viral pneumonia but did not prevent the occurrence of intravascular microthrombosis, which led to her second deterioration. Dexamethasone administration may have temporarily prevented COVID-19 coagulopathy [9]; but the risk of procoagulant events may have increased following its withdrawal, due to the reduction in steroid levels, resulting in mounting intravascular microthrombosis, analogous to the situation that occurs in HLH relapse. In such a situation, coagulopathies may be difficult to diagnose because steroid administration may mask the patient’s fever or other clinical conditions and laboratory changes [15]. Damaged endothelial cells and NETs-containing microthrombi may be the predominant source of cytokines and thrombi after viral clearance. Heparin administration restored the damaged endothelial cells, protected endothelial cells from leukocyte adhesion, and dismantled NETs, which prevented further cytokine release and thrombus formation [18, 19]; and eventually the patient’s D-dimer, ferritin, and sIL-2R levels normalized. We hypothesize that heparin rather than steroid [20] administration, is essential to treat DVT and PE affected by dexamethasone administration.

This report has several limitations. We did not measure coagulation markers around the dexamethasone withdrawal period and prophylactic anticoagulants had not been administered. The risk factors for rebound cytokine production following procoagulant events after treatment completion are unknown; as seen our patient, it can occur in improving, mobile patients. Therefore, careful follow-up of vital signs and coagulation markers during the dexamethasone withdrawal period would be important; this period can be a clinical pit fall period. Use of contrast-enhanced CT to detect coagulation and thrombi and therapeutic heparin administration are useful for PE management. We believe that the causal role of steroid withdrawal in triggering coagulopathy requires further investigation.

## Data Availability

All data generated or analyzed during this study are included in this published article.

## Abbreviations

COVID-19: Coronavirus disease 2019
CT: Computed tomography
DVT: Deep vein thrombosis
HLH: Hemophagocytic lymphohistiocytosis
NET: Neutrophil extracellular traps
PE: Pulmonary embolism
SARS-CoV-2: Severe acute respiratory coronavirus 2
sIL-2R: Soluble interleukin-2 receptor
TRCR: Transcription-reverse transcription concerted reaction
